# Heavy Metals, Their Phytotoxicity, and the Role of Phenolic Antioxidants in Plant Stress Responses with Focus on Cadmium: Review

**DOI:** 10.3390/molecules28093921

**Published:** 2023-05-06

**Authors:** Evgenia A. Goncharuk, Natalia V. Zagoskina

**Affiliations:** K.A. Timiryazev Institute of Plant Physiology, Russian Academy of Sciences, 127276 Moscow, Russia

**Keywords:** heavy metals, metal-induced stress, phytotoxicity, reactive oxygen species, low molecular antioxidants, phenolic compounds, anthocyanins

## Abstract

The current state of heavy metal (HM) environmental pollution problems was considered in the review: the effects of HMs on the vital activity of plants and the functioning of their antioxidant system, including phenolic antioxidants. The latter performs an important function in the distribution and binding of metals, as well as HM detoxification in the plant organism. Much attention was focused on cadmium (Cd) ions as one of the most toxic elements for plants. The data on the accumulation of HMs, including Cd in the soil, the entry into plants, and the effect on their various physiological and biochemical processes (photosynthesis, respiration, transpiration, and water regime) were analyzed. Some aspects of HMs, including Cd, inactivation in plant tissues, and cell compartments, are considered, as well as the functioning of various metabolic pathways at the stage of the stress reaction of plant cells under the action of pollutants. The data on the effect of HMs on the antioxidant system of plants, the accumulation of low molecular weight phenolic bioantioxidants, and their role as ligand inactivators were summarized. The issues of polyphenol biosynthesis regulation under cadmium stress were considered. Understanding the physiological and biochemical role of low molecular antioxidants of phenolic nature under metal-induced stress is important in assessing the effect/aftereffect of Cd on various plant objects—the producers of these secondary metabolites are widely used for the health saving of the world’s population. This review reflects the latest achievements in the field of studying the influence of HMs, including Cd, on various physiological and biochemical processes of the plant organism and enriches our knowledge about the multifunctional role of polyphenols, as one of the most common secondary metabolites, in the formation of plant resistance and adaptation.

## 1. Introduction

Heavy metals (HMs) are among the most widespread compounds on our planet, the composition and content of which regulate physiological and biochemical processes in all living organisms, from microorganisms to human beings [1,2,3]. They participate in natural and manmade cycles of the planet’s ecosystem, engage actively in complexation, and are known to have different toxic effects on living systems [2,4]. Many of the HM molecules are considered persistent pollutants [5].

**Figure 1:**
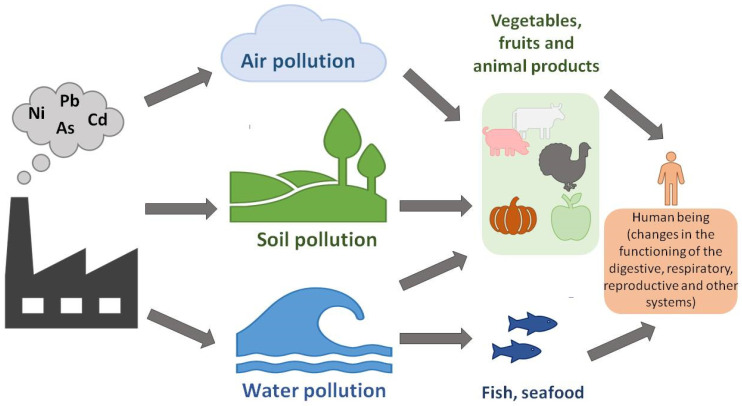
Distribution of heavy metals in the environment.

HMs are always present in the environment; however, humankind’s anthropogenic activities tend to increase their quantities (Figure 1).

This contributes to air and soil pollution, contamination of groundwater, rivers, and oceans, and affects the yield and quality of agricultural, fruit, food, and medicinal crops, as well as human health [3,6].

The HMs include mostly transition metals of the periodic table whose atoms have complex and labile outermost electron shells [5,7]. Their specific characteristic distinguishing them from other metals and metalloids is that they are stable and non-degradable, meaning they persist in the environment and cannot be removed by means of chemical or biological transformation [8]. The UN Global Monitoring Program set up in 1973 only listed three of the most hazardous HM pollutants—lead, mercury, and cadmium (Cd). Another seven metals, copper, tin, chromium, molybdenum, cobalt, and nickel, were later added to the list, as well as three metalloids—antimony, arsenic, and selenium [9]. These HMs are among the most aggressive factors contributing to biosphere pollution, their amounts released into the environment being significantly higher than the scope of their natural occurrence [8]. They occur in the environment in various forms, including as free cations or an array of chemical and physicochemical compounds forming under different conditions and defined by the source of pollution, type of environment, presence of specific organic and inorganic substances, pH of the environment, the HM’s redox potential and other factors [6,10]. It needs to be mentioned that naturally occurring HMs engage in biochemical cycles 100 times less often, on average, than HMs coming from manmade sources of pollution [11,12]. HM pollution of the environment resulting from industrial waste disposal occurs locally, unlike the pervasive pollution by compounds produced from burning fuels (up to 95% in the form of high-dispersed aerosols).

Cd is one of the HMs that are widely spread in the natural environment and toxic for many living organisms [2,10]. The metal was discovered in 1817 by a German physician, Friedrich Stromeyer, as an element found in low concentrations in the natural environment [13]. At present, Cd content in the biosphere has grown significantly in many countries of the world due to its widespread industrial application, its ability to accumulate, and its low decomposition rate [1,5]. The residence time of Cd in the soil is 1 to 3 years, and 2 years in the nearshore sediments, whereas in ocean water, it can stay over 7000 years [14,15].

**Figure 2:**
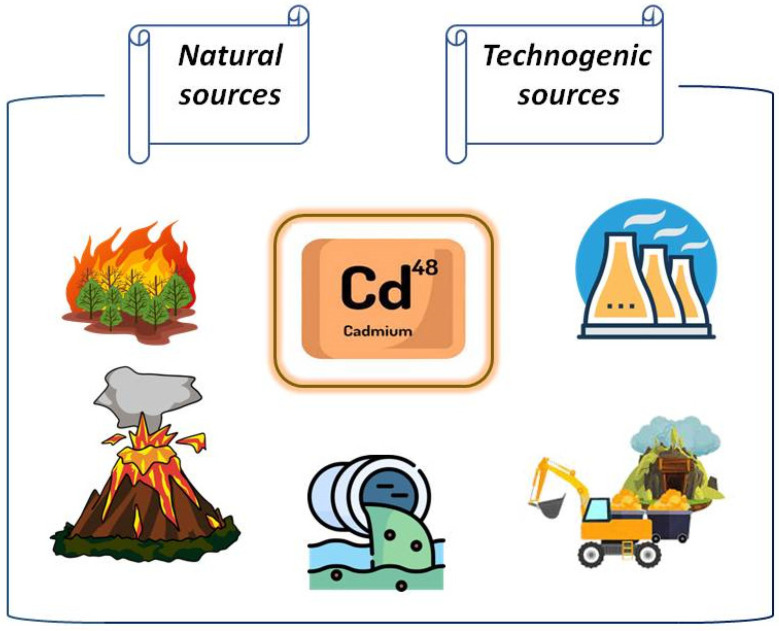
Sources of cadmium (Cd) in the biosphere.

Cd is discharged into the environment by industrial plants producing paints, disinfectants, and alkaline batteries, as well as at non-ferrous metal smelting and ore processing plants dealing with copper, lead, and especially zinc ores (Figure 2).

Heavy metals released in the process of non-ferrous metal smelting (and Cd is a byproduct of zinc production and has an antagonistic effect on zinc) create grave problems for soil health [5]. Additionally, even when toxic waste production sites are located at a significant distance from farming lands, they nonetheless increase both non-carcinogenic and carcinogenic risks for human health related to Cd content in the crops [3,12].

The farming industry also contributes to Cd release into the environment, particularly in phosphate fertilizers that contain Cd as an impurity [16]. The development of a cost-efficient and sustainable production process for removing Cd from phosphates and their derivatives is still a major challenge. This can be explained by the surging price of raw materials and energy and competition among producers of phosphate fertilizers that are valued by farmers for their enhanced effectiveness [17].

Another source of Cd contamination is wastewater, where its content can be very high. This is especially the case in East African countries, India, China, Saudi Arabia, and the European continent [18,19,20]. Additionally, since contamination areas inevitably border densely populated regions, there is a need for proper wastewater treatment and control mechanisms. In this regard, studies of HM removal from the liquid phase are of particular interest, focusing on methods of hydrothermal conversion, pollutant content control, and improved wastewater treatment (including by algae) [21,22].

Cd contamination of the biosphere has toxic effects on plants. It inhibits their growth, destroys their root system, and causes chlorosis and leaf necrosis [7,22]. Among other negative effects are changes in physiological processes, including photosynthesis [23], plant respiration [8], water exchange [24], as well as uptake, transport, and absorption of mineral nutrients [25].

Cd uptake by plants inflicts great harm on human and animal health as it moves along food chains (Figure 1). It damages their respiratory, digestive, and excretory systems [2]. Moreover, Cd accumulates in the human body, and as people grow older, the changes in their metabolic processes become increasingly more pronounced [26].

Thus, Cd and other HMs are always present in our planet’s biosphere, and their content depends on contamination sources, exposure time, the absorptive capacity of the environment, and a number of other factors. Despite the scientific community’s eagerness to study these issues, there are still many unclear aspects related to HM effects on various living organisms, their uptake and distribution in tissues, as well as assessment and understanding of HMs’ tolerance and adaptation mechanisms. This scientific review summarizes the information on the influence of Cd, as one of the most toxic HMs, on physiological and biochemical processes in plants, and their antioxidant system, including phenolic antioxidants as one of the most common representatives of secondary metabolism in plants, which play an important role in adaptation processes.

## 2. Main Plant Groups in Terms of HM Tolerance

Although many of the HMs present in the environment are highly phytotoxic, plants manage to survive by resisting their toxic effects [2]. To some extent, this is the result of their physiological and biological characteristics, specificity to the uptake of certain metallic elements, and successful strategies of adaptation to the toxic effects of these exogenous molecules [4,7].

All plants are presently divided into three large groups based on their sensitivity to HM effects.

*The first group includes plant accumulators*. This group of plants is of great interest to researchers in terms of studying plant response to HM effects and their practical application for phytoremediation purposes [27,28]. The group includes various metallophyte floras that colonize geochemical anomaly areas and have developed, in the process of evolution, constitutive mechanisms of HM tolerance [27]. To help the plants adapt to heavy metal exposure, new ecotypes and populations are formed that have enhanced genetic tolerance to metals [29]. The plants can accumulate HMs in metabolically inert organs and organelles or incorporate them into chelated complexes transforming them into physiologically safe forms [27].

Most plants that are resistant to HM toxicity are characterized by increased absorption of metallic elements, which is accounted for by metal detoxification mechanisms [28]. The discovery of hyperaccumulator plant species, capable of absorbing 50 to 500 times higher concentrations of HMs compared to other plants, spurred the development of phytoextraction technology [30]. There are presently about 400 known hyperaccumulator species (0.2% of all angiosperms), mostly from the families *Asteraceae*, *Brassicaceae*, *Caryo phyllaceae*, *Cyperaceae*, *Cunoniaceae*, *Fabaceae*, *Flacourtiaceae*, *Lamiaceae*, *Poaceae*, *Violaceae,* and *Euphorbiaceae* [31,32]. Due to the mentioned properties, these species are increasingly used for bioremediation of contaminated areas.

There have been instances of HM uptake decrease in plants when HM-resistant ecotypes had lower metal concentrations in their tissues than non-resistant populations. This was registered for *Silene maritime* and *Silene paradoxa*, two species resistant to zinc (Zn) and Cd [4,33,34]; for *Silene cucubalus* and *Silene vulgaris*, a resistance to copper (Cu) [35,36]; and for *Festuca ovina* and *Aster alpines*, a resistance to lead (Pb) and Cd [27]. Reduced metal accumulation in plant cells and tissues can be the result of its intake slowdown and deposition to the root surface though binding to the plant’s slime layers [7,37,38]. This mechanism would have been one of the most effective ways to prevent HM uptake by plants; however, its natural occurrence is extremely rare.

*The second group* is called indicator plants. HM content in them is the same as in the surrounding soil [37,38]. This group of plants can absorb high concentrations of metals and accumulate them in the shoots and leaves, which reflects pollutant concentrations in the soil. The plants can be used as indicators of HM presence and for assessment of soil contamination levels. Phytomonitoring and phytoindication are the primary application fields for these plant species. Bioindicators of Cd, lead (Pb), mercury (Hg), and nickel (Ni) pollution include *Chara baltica*, *Cladophora*, *Coccotylus truncatus*, *Furcellaria lumbricalis*, *Polysiphonia fucoides*, *Stuckenia pectinate,* and *Zanichellia palustris* [39]; among bioindicators of Cd, copper (Cu), lead (Pb), and zinc (Zn) are *Phragmites australis*, *Typha capensis*, *Spartina maritima* [40]; and bioindicators of Cd, cobalt (Co), chromium (Cr), copper (Cu), iron (Fe), nickel (Ni), lead (Pb), and zinc (Zn) include *Patella vulgata* and *Fucus serratus* [41].

*The third group consists of excluder plants.* This type of tolerance strategy is characterized by low HM concentrations in the plant cells despite the high levels in the surrounding soil [42]. The plants of this group block metal transport to the shoot system while accumulating these molecules in the roots. They can be an effective tool for soil stabilization, curbing the further erosion-related spread of HMs and phytostabilization.

Phytostabilization involves HM accumulation in the plant’s root system or in the soil zone adjacent to the roots—the rhizosphere [43]. This alters the chemical composition of the soil solution and activates the processes of absorption and precipitation of these molecules by the soil absorbing complex (SAC) [44]. Moreover, the redox enzymes of plants facilitate HM transition into less toxic forms during phytostabilization [43]. Excluder plants are actively grown in contaminated areas to prevent pollutants from migrating to groundwater reservoirs [45].

Thus, considerable differences in plant tolerance to HM toxicity are derived from their physiological and biochemical characteristics as well as the mechanisms they use to interact with metal molecules and absorb them. The investigation of these mechanisms and optimization strategy development for plant organisms are among the most important and topical areas of scientific research.

## 3. Inactivation of HMs in Plants

The absorption and deactivation of HM contaminants after their uptake by plants are explained by the action of two types of mechanisms found in plants—constitutive mechanisms, which are part of plant homeostasis, and adaptive mechanisms, triggered by exposure to stress [23,24]. These mechanisms contribute to three main processes taking place in plants.

*1. Limitation of HM uptake by plants and individual plant organs.* It proceeds with the help of chelators—compounds found in the plant’s root exudates that are capable of binding to toxic metal ions. Organic acids, amino acids, and phenolic compounds are examples of chelating agents [7,24,37]. For example, the root exudates of sorghum and tomato plants contain malic and oxalic acids, which reduce the plant’s sensitivity to the toxic effects of HMs [46]. Plant genotypes that had malic acid in their root exudates exhibited tolerance to high concentrations of aluminum (Al) in the soil solution [47]. Under nickel stress, histidine and nitrogen oxide acted as chelating agents toward the contaminants in the xylem cells of the plant genus *Alyssum* [48]. An increase in the concentration of these compounds was also registered for other plant species when exposed to various HMs [49].

Changes in the pH of the rhizosphere are observed in the presence of HMs, and Cd in particular, for cereal grass cultivars—its acidity tends to increase, resulting in reduced uptake of HMs by the plant’s root system [50]. The increase in the medium pH is caused by the effects of various root exudates that bind to metals and precipitate them to the apoplast, blocking their entry into the cell [51]. Some wheat species have an effective secretory system and can grow under HM toxicity stress [47].

*2. Inactivation of the HMs entering the plant.* This process involves a more widely spread mechanism of coping with metal toxicity [22,28]. There are basically two ways how plants carry this out: one, by forming insoluble inorganic compounds, and two, via intracellular binding to organic substances to form insoluble or soluble complexes with low physiological activity, as well as strong complexes with specifically induced substances.

In the first case, particles containing HMs in the form of phosphates, silicates, and sulfides are deposited in the cell. Thus, insoluble copper compounds were found in spinach cell cytoplasm when the surrounding environment contained toxic quantities of this metal [52]. Studies have found microcrystalline zinc-containing particles of low solubility in the epidermis of *Silene cucubalus* as well as rhizodermis and pericycle cells of *Allium cepa* [53,54]. Dense zinc-containing globules were localized within vacuoles of root cortical cells of *Deschampsia caespitosa* [55]. The formation of inorganic inclusions and granules is a long-term HM detoxification mechanism employed by plants.

Inorganic insoluble complexes are typically formed in the cell walls of plants, where most of the absorbed HMs are accumulated. They can be viewed as an HM-immobilizing compartment protecting the cytoplasm and cellular organelles from their toxic effects.

In rice species, the capacity for Cd retention in the cell wall is based on pectate formation occurring when metals interact with the cell wall pectins containing high concentrations of uronic acid and pectin methyl esterase (PME) with pronounced activity [56]. Apart from pectic substances, HMs also bind to cell wall polysaccharides.

Besides HM binding and isolation capacity of various chelators, there are data confirming changes in the physicochemical properties of cell walls when the plant is exposed to HMs, for example, an increase in cell wall lignification and suberin deposition observed in both in vivo and in vitro experiments [57,58].

The capacity of a plant organism for resisting HM effects is not limited to the barrier function of its cell walls that have low selectivity for HMs. There are other protective mechanisms that are employed when higher concentrations of pollutants enter plant cells from the contaminated environment. One is the cell membrane (plasmalemma), known to activate its barrier function under stress; the cytoplasm is yet another example as the HM binding takes place there, and the metal complexes and free ions are transported to vacuoles [59,60]. For example, the formation in the cytoplasm of soluble slow-dissociating complexes that are stable compounds of metal and citric or malic acids, and their entry into the tonoplast, further release of the metal, formation of different complexes involving citrates and oxalates, and possible transfer to the vacuoles, can also be considered an HM tolerance mechanism [51].

*3. Changes in the metabolic pathways as a stress response of plants to HM toxicity.* This is yet another defense mechanism devised by plants to help them to survive and adapt. Intensification of biosynthesis of high-molecular-weight compounds, polyamines, metallothioneins, and stress proteins, as well as changes in the hormonal balance of plant tissues, were observed in stressed plants [7,37].

The formation of metal-binding compounds such as phytochelatins (PCs) and metallothioneins (MTs) is a specific response of plant cells to HM toxicity [23,52]. Phytochelatin-dependent detoxification of HM ions and subsequent PC-metal complexes within vacuoles are very important processes for plant survival under anthropogenic stress [58,61,62]. This protective mechanism helps to avoid pollutant binding to physiologically important proteins and facilitates metal transport into the vacuoles. Post-translational protein modifications, particularly protein degradation by the ubiquitin-proteasome system (UPS), are vitally important for cell homeostasis as well as plant interaction with the surrounding environment and plant response to HM stress [63]. It has been shown that the ubiquitin ligase gene participates in the regulation of tomato plant tolerance to Cd [64].

The favored ligands for HM binding are thiols present in glutathione and PCs [65]. An increase in glutathione content is viewed as a means of boosting the metal-binding capacity of plant cells, as well as strengthening cellular defense against the harmful effects of oxidative stress. However, increasing biosynthesis alone seems insufficient to achieve even a small enhancement of the plant’s HM tolerance capacity. The decisive factor here is a vacuolar transporter (YCF1) of glutathione conjugates: glutathione synthetase (GS); the overexpression of this gene confers higher capacity for HM accumulation in plant cells, as was demonstrated for *Arabidopsis* plants [66].

Along with PC accumulation, a decrease in the glutathione pool was observed, which is accounted for by its role in PC formation [23,58]. Such mobile electron donors as glutathione regulate protein activity in the metabolic and signaling pathways through redox processes involving amino acid residues, thus forming protein interaction networks [67,68]. Mobile electron donors and their related enzymes are often viewed as part of integrative redox systems.

Pollutant tolerance depends on the capacity for active HM binding, in particular Cd, by non-protein thiols, which was observed in the roots and shoots of maize, rice, barley, and *Setaria viridis* [69]. Glutathione is the precursor of PCs, and phytochelatin synthase (PCS) is known to catalyze their production [68]. PCS is a constitutively expressed enzyme that is subject to post-translational activation by metals. The formation of thiolates (such as Cd–GS_2_), which act as high-affinity substrates for the enzyme, seems to be sufficient for its activation. PC synthesis is presumed to be one of the main factors of Cd tolerance in plants wherein most of the metal is chelated in the roots (with the exception of hyperaccumulator plants). Researchers believe that Cd-binding by non-protein thiols is exceptionally tight and chemically stable and, therefore, can be considered one of the most important HM detoxification mechanisms, which can be different depending on the plant species [66,69].

It has also been reported that under HM stress, including Cd, the mitogen-activated protein kinase (MAPK) cascade transfers the signals perceived by cell membrane surface receptors to cells by means of phosphorylation and dephosphorylation and targets various effector proteins or transcriptional factors so as to evoke a stress response. Various signal molecules can activate the MAPK cascade through differentially expressed genes, leading to the antioxidant system activation in order to regulate plant responses to HMs. Transcriptional factors, located downstream of MAPK, are key to regulating plant responses to HMs and improving HM tolerance and accumulation in plants. An understanding of how HMs activate the expression of genes related to the MAPK cascade pathway and then phosphorylate the transcriptional factors can help us develop a regulation network and investigate molecular mechanisms underlying plant tolerance to metal-induced stress and capacity to accumulate HMs [70].

## 4. Cd, Its Uptake by Plants and Interaction with Other Metals

Cd is one of the most toxic and highly mobile pollutants capable of accumulating in different organs of plants [71]. The threshold of phytotoxic concentration of Cd varies across plant species and ecotypes depending on the metal’s concentration and the time and routes of the plants’ exposure to it.

A number of plants have been identified as Cd hyperaccumulators that demonstrate positive correlations between Cd concentrations and their morphological parameters. Such plants are capable of accumulating up to 100 mg kg^−1^ dry weight (DW) (0.01%) of Cd in the shoot, which is greater by a factor of 100 than Cd accumulation rates in plants not considered hyperaccumulators [42,43]. Some plants are capable of accumulating even more Cd. For example, *Arabis gemmifera* can accumulate up to 6000 mg kg^−1^ dry weight (DW) of Cd when cultivated in Cd solution. The bioconcentration factor (BCF) of Cd in *Chromolaena odorata* [72,73], *Chara aculeolata* and *Nitella opaca* [73] was found to be greater than 1000.

The plant response to Cd stress depends on the Cd concentration plants are exposed to. For instance, exposure of *Alternanthera bettzickiana* (*Amaranthaceae*) plants to 0.5 mmol and 1 mmol Cd concentrations resulted in their increased growth, increased biomass, increased levels of photosynthetic pigments, and increased activity of various enzymes such as superoxide dismutase (SOD), catalase (CAT), and peroxidase, whereas exposure of the same plants to higher concentrations of Cd, such as 2 mmol, resulted in a decrease in all the same parameters [74].

Cd enters plants mostly through absorption by the roots [25], which is why it affects the roots more compared to the shoots [69]. Inhibition of root elongation has been shown to be one of the earliest and most distinct symptoms of Cd toxicity [59]. This effect has been attributed to associated decreased water content in root tissues, depolymerization of microtubules of the cell cytoskeleton, and the formation of chromosome aberrations, which result in lower mitotic activities of meristematic cells [75]. Cd exposure has been seen to cause the formation of a more compact root system in plants due to inhibition of the main root elongation and proliferation of lateral roots that are less affected by Cd pollution [23,27]. Cd stress has been noted to result in greater root diameter in trees [76], which is associated with an increase in the size of parenchymal cells and cortical tissue expansion [77]. Exposure to Cd has been reported to inhibit root growth in nitrogen-fixing plants as well as alter their root structure by way of significantly decreased (up to 70%) root nodule formation [76]. Additionally, the major visible symptoms of Cd-induced toxicity in plants included root browning [59,78]. Thus, the observed changes in morphometrical and morphological parameters of roots have been proposed to be possible indicators for evaluating the toxicity of Cd in plants.

Chlorosis, necrosis, and drying are toxic symptoms of Cd stress in the foliage of plants [7]. According to Shrivastav P. et al. [48], the concentration of Cd in the leaves in excess of 5–10 mg kg^−1^ DW is toxic and causes the abovementioned changes in plant morphology. Recent research indicates that Cd is transported from the soil only into the growth area of the leaf blades while not being readily remobilized to other plant organs or tissues, the way it happens with some biogenic elements [24,59]. It has been reported that Cd tends to accumulate more and cause more visible toxicity signs in older leaves than in younger ones [79]. It has also been noted that Cd relocation to leaves by xylem occurs at a much slower rate than its potential translocation via phloem as part of compounds [25].

In addition to soil contamination, air pollution can also be a source of Cd accumulation in plants through foliage, with the subsequent emission of some Cd through transpiration. In cases of significant air pollution, foliar concentrations of Cd can be even higher than Cd root uptake [80]. Uptake of Cd together with aerosol or hydrosol particles of technogenic origin occurs through the gas exchange mechanism facilitated by the leaf surface as well as its substomatal cavities and mesophyll airspaces [81]. In such cases, Cd is not absorbed into the symplast.

Thus, Cd is predominantly accumulated in plants through the roots wherever the pollutant is present in the soil. The contribution of foliar uptake of Cd is relatively low and of significance only in the areas where the atmosphere is highly polluted by human activities.

Cd toxicity in plants is defined by its interaction with important macro- and micronutrients such as zinc (Zn), iron (Fe), calcium (Ca), potassium (K), manganese (Mg), copper (Cu), and silicon (Si). At the same time, Cd competes with other divalent cations of metals, metalloids, and transitional elements for complexation with soluble organic ligands. The presence of such competing ions in the soil can lead to decreased soil Cd availability to plants [59,82]. It has also been reported that the use of fertilizers containing K, Mg, and Si, as well as iron, aluminum, and manganese oxides, significantly decreased the soil bioavailability of Cd due to ion-exchange reactions and adsorption processes [83]. The presence of Cd in the nutrient solution has been noted to inhibit the translocation of other metals from roots to the aerial parts of the plants, as well as to affect the formation of phytochelatins. It has been observed that responses to excessive Cd content can vary among plant species. Thus, the same Cd concentrations have been associated with decreased levels of Cu root uptake by ryegrass, corn, cabbages, and clovers, increased Cu root uptake by rice, and unchanged levels of Cu root uptake by pumpkins and cucumbers [84]. Disturbance of ion homeostasis can also be caused by the loss of ions by the roots, such as K ions, due to altered activity of membrane enzymes or membrane damage [50].

The exogenous Ca had an antagonistic effect on Cd accumulation in plants when introduced with a nutrient solution via calcium channels [83]. Cd toxicity has been reported to hinder signaling processes in plants leading to stomatal closure, declining transpiration rate, and overall stunted growth. In particular, inhibited stomatal closure has been reported in tobacco plants [85] and mung bean (*Vigna radiata*) [86].

Cd phytotoxicity has been found to be in direct relationship with the Zn content in plants due to Cd’s ability to replace Zn in the metal–protein complexes of enzymes [4]. In most cases, low Zn content in plants has been observed to induce higher Cd absorption rates, and the other way around [87]. With comparable concentration levels of these two antagonist metals, however, Zn uptake exceeded Cd uptake at least twofold [88]. In some plants capable of hyperaccumulating Cd in the shoots, Cd accumulation was not inhibited by Zn, suggesting that Zn has no influence on the absorption and translocation of Cd in the underground and aboveground plant organs [89,90]. There is evidence that high concentrations of Zn and Cd in the soil can lead to increased Cd concentration in the aboveground organs of plants [91]. All this is not only indicative of the antagonistic relationship between Cd and Zn, but also suggests that there is a specific and efficient Cd transport system in plants [59,90].

Speaking of other essential elements and their interactions with Cd in plants, it is important to note the ability of some of them, such as Si, to render active heavy metal chemical forms inactive through complexation, deposition, and conversion of metallic ions into non-phytotoxic forms in less biologically active tissues, such as epidermal or storage tissues [59,92].

There have been reports about the interaction of Cd and Fe. Fe deficiency was observed in the roots of *Amaranthus mangostanus* plants exposed to high-concentration Cd solution [93]. At the same time, hyperaccumulator plants have been reported to increase their absorption rates of Cd and Fe when exposed to both [94].

Inhibited enzyme activity has also been reported due to Cd exposure [7,23]. This is associated both with Cd binding to the functional SH-groups in proteins and the disrupted ionic balance caused by decreased absorption of Zn, Fe, Cu, and other metals, as well as their impaired transport [7,72]. Cd-induced inhibition of phytoenzymes, in turn, reduces K uptake in plants, loss of K by cell organelles, and stunted translocation of Fe to leaves and fruit [24,37].

In general, Cd stress affects the uptake of many essential elements (Fe, Mg, Si, Zn, Cu, Ca, etc.), and in most cases, Cd is antagonistic towards them, competing for membrane transport. Additionally, Cd has been reported to affect the translocation of elements absorbed by the plant from the soil solution [59,89,90]. The processes of Cd uptake and absorption, its competition with other metals, and the resulting uptake of either metal in each case largely depend on the growing conditions of plants, their species, and metal accumulating capacities.

## 5. The Effect of Cd on Physiological and Biochemical Processes in Plants

Excessive Cd accumulation in plants can affect photosynthesis [23], respiration [8,24], water exchange and uptake, transport, and absorption of mineral nutrients [25]. Visible symptoms of Cd toxicity, such as chlorosis of leaves, root rot, wilt, etc., usually develop when its total concentration in the soil exceeds 8 mg/kg, its bioavailable concentration exceeds 0.001 mg/kg, or Cd concentration in plant tissues reaches 3–30 mg/kg [95,96]. The mentioned changes are the result of disrupted or altered physiological and biochemical processes of the plant organism we will discuss in this paper.

*Water exchange.* As for Cd effects on such an important physiological process as water exchange, it has been noted that Cd toxicity changes the water status in plant tissues by reducing their water content, stomatal conductance, transpiration rate as a result of cell vacuolization, ‘shrinkage’ of intracellular space, reduction in the chloroplast number and increased cell size [97]. It has been shown that Cd alters the plasma membrane permeability, including to water, which results in the reduction in water content in plant tissues and water balance disruption [23]. Thus, plants colonizing ecologically challenged regions are characterized by the lower water content of tissues and a reduced transpiration rate, which affects the thermal regime of leaf tissues [98]. In many cases, changes in the plant’s water status can lead to a number of physiological modifications, reducing its osmotic regulation effectiveness and cell wall elasticity, and damaging the water absorption capacity of the root [99]. The decline of water absorption capacity of the plant’s root system is primarily caused by inhibition of its formation, linear growth arrest of the taproot, and the slowdown of photoassimilate transport from the shoots to the roots. This also accelerates root tip necrosis, increases cell lignification and suberization, and drives up the content of abscisic acid, causing stomatal closure [100,101].

Cd was also shown to inhibit the process of seed swelling and, therefore, to reduce their germination speed. Reduced water content in the sprouts resulted in a water deficit at the early stages of plant ontogenesis, which was observed for *Medicago sativa* and other crop plants [102], pea [103], and rice [104]. Cd-induced inhibition of seed swelling, smaller seed size, and lower speed of germination were common manifestations found in *Ocimum basilicum* [105].

*Photosynthesis.* The photosynthetic system is one of the most sensitive indicator systems of plant physiological status. Cd stress severely affects such parameters as photochemical efficiency and photosynthetic intensity, as well as chlorophyll content in plants [106].

It has been established that Cd inhibits photosynthetic processes significantly, reducing the chlorophyll content in plants and destabilizing chlorophyll–protein complexes, which leads to photosynthetic apparatus damage. A decline in the photosynthetic pigment content under metal stress was mostly observed in the total chlorophyll content, especially chlorophyll *a*, as compared to carotenoids [107]. These changes are thought to be the consequence of inhibited biosynthesis of chlorophyll and its degradation [23]. It has been reported that Cd also affects the chlorophyll content indirectly by inducing micronutrient deficiency. Thus, the symptoms of Cd toxicity in plants are often similar to iron deficiency manifestations. Cd causes disorganization of chlorophyll–protein complexes that are combined or substituted with newly formed Cd–chlorophyll complexes. Cd stress caused a decrease in iron citrate transporter expression in the xylem parenchyma cells of plant roots, disrupting iron trafficking to the shoots [108].

It is also known that Cd causes bond degradation within thylakoid pigment–multiprotein complexes, disrupting their donor–acceptor interactions (coordinate bonds) and affecting chloroplast stability. It has been observed that Cd affects the light phase of photosynthesis by disrupting electron transport mainly related to photosystem II (PSII), which is the result of Calvin cycle enzyme inhibition, changes in thylakoid membrane structure, damage to plastoquinone pool as well as decreased activity of ferredoxin-NADP+ reductase (FNR), reduced quantum efficiency of PSII and electron transfer rate [109]. However, Cd effects on plant photosynthetic performance are species-specific and largely defined by its concentration in the soil solution [23,110].

*Respiration.* Another important energy-producing process in plants is respiration. Cd stress was shown to inhibit respiratory enzyme activity, such as succinate dehydrogenase, succinate cytochrome reductase, cytochrome oxidase, etc., affecting respiratory intensity in plant cells. The negative effect of the metal on the plant respiratory process is manifested through the damaged activity of the Krebs cycle enzymes and electron transport chain function and is one of the reasons for Cd-induced seed germination damage [109]. It has been reported that oxygen absorption by tobacco roots and isolated cells decreases in Cd presence [111]. Respiratory function intensification was reported in barley and oats under Cd exposure, which is accounted for by the increased activity of several respiratory enzymes [50,112]. However, high Cd concentrations produced the opposite effect [110]. Thus, higher doses of pollutants caused a decline in respiration intensity in rice, maize, and barley plants, which resulted from activity inhibition of the key enzymes of glycolysis, the pentose phosphate pathway, and the Krebs cycle [113,114].

*Nitrogen metabolism.* Cd affects nitrogen assimilation, a process responsible for stable growth and reproductive performance of higher plants [115]. It has been shown that Cd inhibits nitrogen metabolism by hindering the activity of nitrate and nitrite reductase, two enzymes participating in nitrate ion assimilation [116]. Cd also inhibited the activity of enzymes involved in ammonium ion assimilation, which hinders root nodule formation in leguminous plants, in particular *Lupinus albus*, as a result of sucrose synthase suppression and lower nitrogen fixation [117].

*Enzyme activity regulation*. As mentioned above, Cd toxicity affects enzyme activity [118]. This can be the result of its non-specific interaction with various complexing protein groups. The protein molecule can change its configuration and ‘open up’ new active groups leading to the modification of its properties. Moreover, HM ions, and Cd in particular, are capable of binding to active sites of enzymes and blocking them, as well as displacing the metal ion, which is essential for enzymatic activity, from the active site of metalloenzymes. The inactivation constant for HMs has a broad range, and for Cd ions, it is 10^−6^–3 × 10^−5^ M. Cd interaction with enzymes is rather complicated as Cd ions are not needed for enzymatic activity in plants. Thus, studies of HMs’ toxic effects on plants have shown an increase in enzymatic activity at low metal concentrations, which reflects the general plant response to the presence of toxic ions in the environment. However, in the case of higher concentrations of metal ions, with more of them getting to coordination sites, they have mostly inactivating effects [7,54].

**Figure 3:**
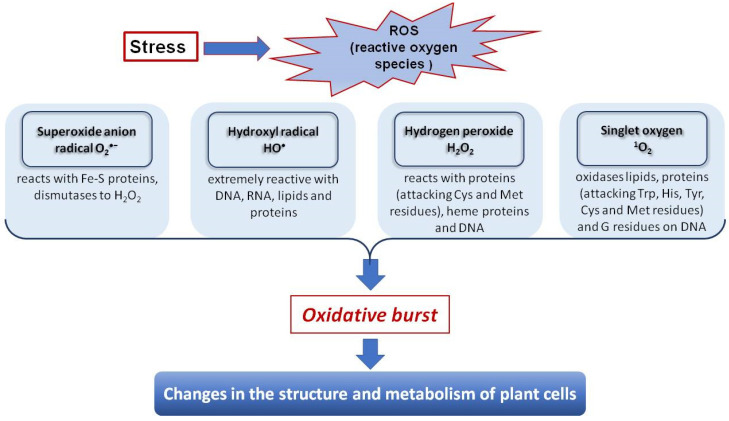
Stress as an important factor leading to the accumulation of reactive oxygen species in plant cells and the consequences of their action.

*Reactive oxygen species (ROS) generation*. It is known that ROS molecules are produced during respiration and photosynthesis in all plant cells (Figure 3).

In stress conditions, including Cd stress, the number of ROS produced by plants increases, such as superoxide anion radical O_2_^•−^, hydroperoxyl radical HO_2_^•^, hydroxyl radical OH^•^, hydrogen peroxide H_2_O_2_, and singlet oxygen ^1^O_2_ [119]. Free radical accumulation leads to the so-called ‘oxidative burst’ affecting metabolism, initiating pathological processes, causing necrotic lesions in vegetative and generative plant organs, and even plant death [120]. Increased ROS formation in the cells results in the oxidation of lipids, carbohydrates, and proteins, DNA and RNA damage, and cytoskeleton disorganization [119,121].

It has been shown that Cd stress affects gene expression, as observed in raps and mint plants [122,123], altering protein biosynthesis. It can induce or inhibit enzyme activity and launch lipid peroxidation (LPO) processes, increasing ROS content in plant cells [60,120].

Thus, a significant increase in LPO activity, as an indicator of the plant stress response, and high levels of H_2_O_2_ accumulation were observed in Asian rice (*Oryza sativa*) and common pea (*Pisum Sativum*) sprouts under metal-induced stress. Sprouts of thale cress (*Arabidopsis thaliana*), wheat (*Triticum vulgare*), cucumber (*Cucumis sativus*), quinoa (*Chenopodium quinoa*), and basil *(Ocimum basilicum)* exhibited a significant increase in H_2_O_2_ and O^2•−^ levels and malondialdehyde (MDA) content when exposed to Cd [105,124]. It has also been reported that the sprouts of adzuki bean (*Vigna angularis*) exhibited increased lipoxygenase activity when exposed to excess concentrations of Cd, while field mint *(Mentha arvensis*) showed increased levels of H_2_O_2_ and LPO byproducts [115,125,126]. In general, it has been demonstrated that ROS act as signaling molecules and mediate different plant cell responses to metal-induced stress.

**Figure 4:**
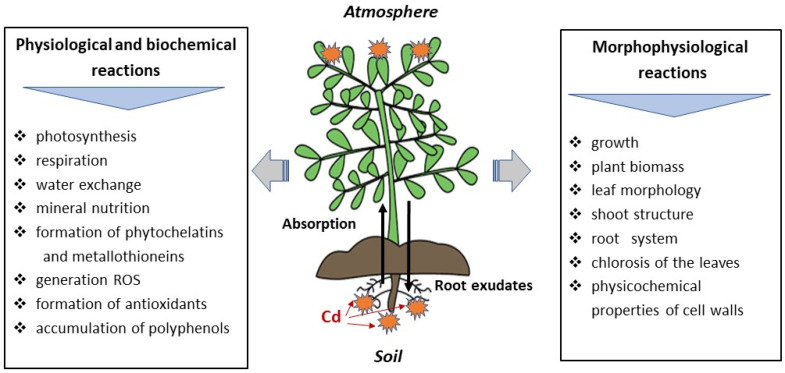
The penetration of cadmium into plants leads to changes in its morphology and metabolism.

Thus, it can be said that almost all physiological processes occurring within the plant organism are affected by the toxic effects of Cd (Figure 4).

However, plants continue to grow and colonize Cd-contaminated areas employing various adaptation mechanisms [118].

## 6. Phenolic Antioxidants (AO) and Their Role in Stress-Coping Strategies

Investigation of plant responses to different types of stress is one of the most dynamically growing areas of biological sciences, which is confirmed by a significant increase in the number of publications, especially in recent years [52,58]. Acute interest in this topic is encouraged by the environmental changes taking place on our planet, mounting anthropogenic pressure, and the increasingly broad use of plants and plant-based products for human life support and health protection [127,128].

**Figure 5:**
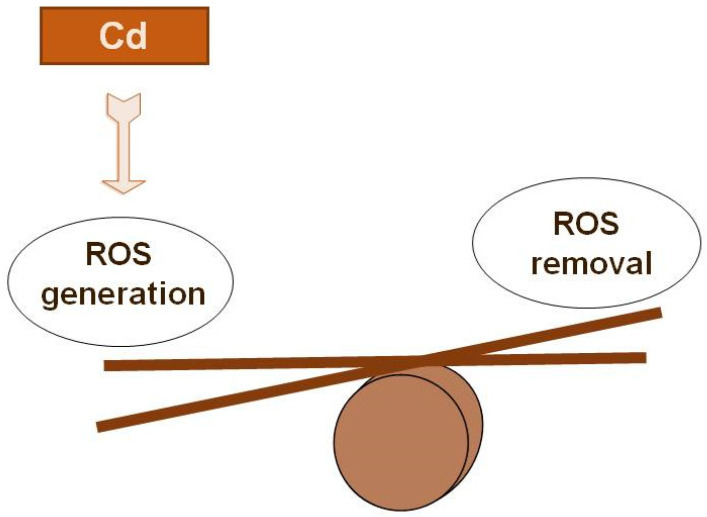
Effect of cadmium (Cd) on the balance of reactive oxygen species (ROS) in plant cells.

*ROS and antioxidants (AOs).* It is known that environmental stress induced by various factors, including Cd as one of the most toxic HMs, affects the metabolic homeostasis of the plant organism [23,24]. This is the result, to a great extent, of an imbalance between ROS generation and removal (Figure 5).

ROS are highly reactive chemicals that are considerably cytotoxic to all types of cells and cell formations [7]. An increase in ROS production in cells leads to oxidative stress [129] and, as a result, to various pathological processes and plant diseases, necrosis of the vegetative and generative plant organs, and even plant death [130]. At the cell level, ROS activities cause nucleic acid damage through deoxyribose oxidation; breaking of peptide bonds; initiation of the LPO process leading to increased membrane viscosity and disrupted diffusion; and accumulation of damaged and self-assembling proteins. High levels of ROS can cause apoptosis, or programmed cell death [120,129,131].

ROS can be produced in many plant cell compartments, including chloroplasts, mitochondria, peroxisomes, and plasma membranes [120]. In the chloroplast, the chlorophyll pigments absorb light quanta and become excited to their triplet state; and if the triplet chlorophyll is not quenched efficiently, charge recombination occurs, producing excited molecules of singlet oxygen ^1^O_2,_ which diffuse outside the chloroplast to reach the cell wall and cytosol [126]. The superoxide anion radical O_2_^•−^ reacts with H^+^, producing the next free radical, hydroperoxyl radical HO^2•−^, which is far more stable and reactive and can easily penetrate biological membranes. Similarly, H_2_O_2_ can be produced through the dismutation of O^2•−^/HO ^2•−^ [132]. Chemically, H_2_O_2_ acts as a weak acid that is highly diffusible and stable and could cross the plasma membrane via aquaporins [121,132]. Another important ROS, OH^•^, can be produced by the Fenton reaction, hydroperoxide activity in sunlight, and inner-sphere electron transfer [126]. It should be noted that ROS conversions in plant cells (scavenging and neutralization) are performed by AO activity [118,119,126]. These metabolites are present in lower concentrations compared to the oxidized substrate and can effectively slow down or inhibit its oxidation.

AO is divided into enzymatic and non-enzymatic, or high-molecular-weight and low-molecular-weight. The former group is represented by superoxide dismutase, catalase, various peroxidases, and other enzymes, while the latter include low-molecular-weight compounds such as glutathione, ascorbic acid, carotenoids, tocopherols, and phenolic compounds [67,133]. Today, phenolics generate a lot of scientific and practical interest as a component of the plant AO system [134]. It is known that they are capable of interrupting oxidation reactions occurring via the free radical chain mechanism, acting as ‘traps’ for ROS, and can also chelate heavy metals and inhibit metal-catalyzed free-radical oxidation reactions [126,135].

*Phenolic compounds: structure and properties.* Phenolic compounds are an important class of secondary metabolites found in plants. Polyphenols are formed in almost all plant cells. However, they perform a variety of functions, one of which is plant protection against abiotic and biotic stressors [136,137].

Phenolics represent a heterogeneous group of molecules that have different structures, chemical properties, and biological activity [138]. They all have in common an aromatic ring bearing one (simple phenolic compounds) or more (polyphenols) hydroxyl groups, attached directly to a carbon atom in one (hydroxybenzoic and hydroxycinnamic acids) or more (flavonoids) benzene rings.

Most representatives of this diverse group of specialized metabolites are easily oxidized, which results in the formation of highly reactive intermediates such as semiquinone radicals or orthoquinones [138,139]. They inactivate free radicals, thus protecting plant cells against ROS. The AO activity of phenolic compounds depends on the number of OH groups in their molecules [140,141]. Thus, compounds with three or more hydroxyl groups have high AO activity. It is also essential that these groups be located at the C-3′ and C-4′ positions. Additionally, the 2,3-double bond in conjugation with a C-4′- oxo functional group or the presence of OH groups at the C-5′ and C-3 positions with a carbonyl group enhance the reactive capacity even more. Phenolic compounds are very diverse and classified according to their structure: from simple (hydroxybenzoic acids) to high-molecular-weight polymer molecules such as hydrolyzable and condensed tannins (proanthocyanidins) [136]. These secondary metabolites are generally found in plants in conjugated form rather than free, for example, with one or more sugar moieties linked by β-glycosidic bonds to the OH group (*O*-glycosides) or a carbon of the aromatic ring (*C*-glycosides). The sugar bonds can be monosaccharides, disaccharides, or even oligosaccharides, the most common being glucose, galactose, and rhamnose [127].

**Figure 6:**
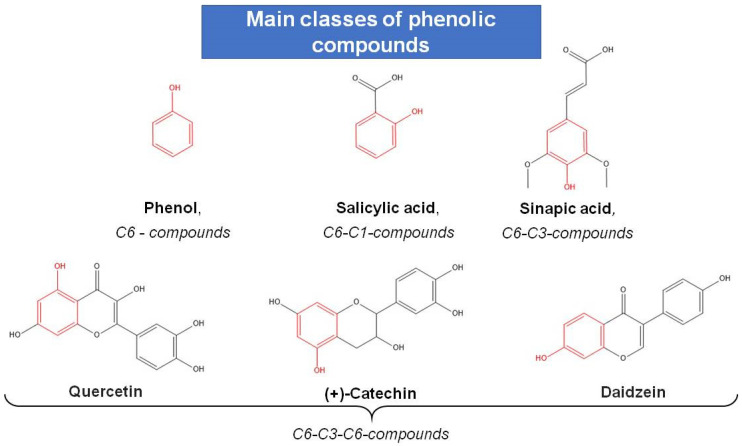
Structural formulas of the main classes of phenolic compounds (from PubChem).

Phenol is the simplest phenolic compound which has one aromatic ring and one phenolic hydroxyl group. All the other phenolic compounds have a more complex structure (Figure 6).

Phenolic compounds with one aromatic ring include hydroxybenzoic acids such as vanillic, salicylic, protocatechuic, and gallic, as well as phenylpropanoids, including hydroxycinnamic, sinapic, and caffeic acids [138,139]. The formation of these metabolites in various plant species was discussed in a number of reviews. According to research data, the number of plant polyphenols exceeds 10,000 [139,142]. Additionally, new compounds continue to be identified due to the broadening of research methodology and active use of high-performance chromatography, mass spectrometry, and other techniques [143]. The processes of hydroxylation, methylation, glycosylation, and acylation of the two benzene rings, A and B, contribute greatly to the diversity of phenolic structural modifications.

Among the most structurally diverse and widespread phenolic compounds are flavonoids—representatives of the largest class of plant polyphenolic compounds with a 15-carbon basic skeleton (C3-C6-C3) that exhibit high AO activity [142]. Over 5000 flavonoids have been identified to date [144]. Their molecules contain two benzene rings connected by a 3-carbon linking chain and a various number of hydroxyl groups (Figure 6).

Despite structural similarities, flavonoids can differ in their properties depending on the positions of the carbon ions to which hydrogen substituents (-OH, -OCH_3_, -CH_3_) are attached, the presence of asymmetric carbons, and the oxidation (or reduction) level of the 3-carbon chain.

**Figure 7:**
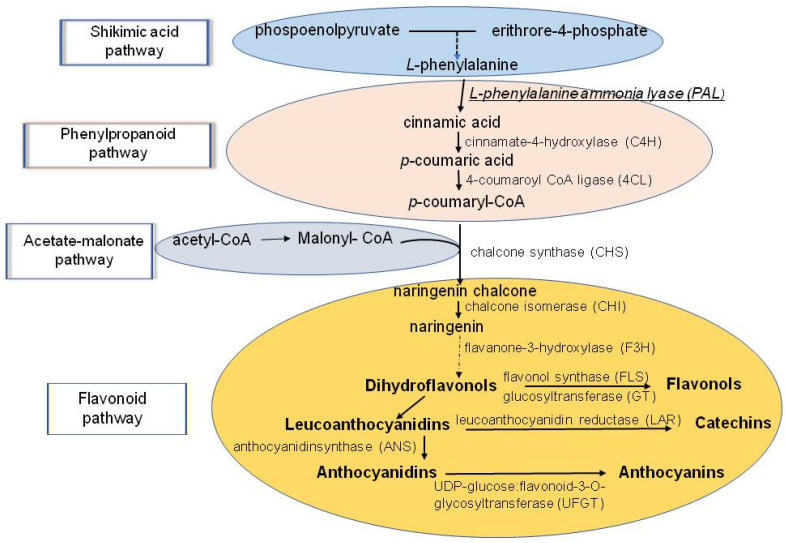
The biosynthesis pathways of phenolics in plants.

Significant progress has been achieved in the research of phenolic compound biosynthesis [145,146]. Phenolics are synthesized through two metabolic pathways—the shikimic acid pathway and the acetic acid pathway (also called acetate-malonate or polyketide) (Figure 7).

All the metabolites, enzymes, and genes involved in this process have been studied [137,147].

*Reactive oxygen species (ROS) and phenolic bio-AOs.* Phenolic compounds are known to neutralize stress-induced changes in plants and modulate the ROS signaling cascade, which forms the basis of their regulatory functions [132,138]. This effect is based on their non-specific redox reactions with small molecules, radicals, and ions [137,144]. It is defined by polyphenols’ capacity to directly interact with free radicals and remove them from the sites of generation [132]. This is performed with the help of preventive and antiradical mechanisms [148]. The preventive mechanism inhibits processes leading to the formation of initiating radicals, while the antiradical mechanism involves AO compounds ‘intercepting’ radical anions and hydroxyl radicals, inhibiting the free radical oxidation reaction chain or interrupting the generation process that has already started.

Reactions between phenolic compounds and free radicals proceed at a very high rate and are accompanied by the formation of phenols of phenoxy radicals as reaction intermediates. Then, these unstable compounds can become derivatives of the parent phenolic compound or enter another cycle of redox reactions [148]. The phenoxy radical reactivity and the structure of compounds it transforms into depend on the structure of the original molecule as well as the reaction conditions.

As previously noted, one of the reaction mechanisms occurring between flavonoids and ROS (superoxide anion radical) is the one-electron reduction in the superoxide anion leading to the generation of hydrogen peroxide [149]. Flavonols are the most potent reducing agents that quench superoxide, in particular quercetin, as it bears a free catechol moiety. It has been established that quercetin and its derivatives, as representatives of the bioflavonoid class, play an important role in the adaptive reactions of plant organisms due to their capacity for ROS signal transduction, which is seen as their priority function [150].

It is known that phenolic compounds are involved in plant cell photoprotection mechanisms, and their biosynthesis is significantly activated by photosynthetically active radiation [151,152] and also when the redox equilibrium of photosynthetic reactions is upset [153,154]. It should be noted that not only flavonoids but also phenylpropanoids, including hydroxycinnamic acid derivatives, can absorb solar UV-B radiation [155].

The AO activity of phenolic compounds is also explained by their high metal-chelating capacity for metals that induce oxidative stress, protecting the cells from its effects [156,157,158]. This capacity is based on the presence of functional groups—one carbonyl and multiple hydroxyl groups [159]. Chelation of metal ions by some flavonoids (through the carbonyl group or π-electrons of these molecules) was successfully applied for the synthesis of anisotropic Au nanoparticles and quasispheroidal Ag nanoparticles from *Lawsonia inermis* [158]. Phenolic compounds were reported to participate in metal ion reduction by converting internal ketones to carboxylic acids [157,160]. This effect is supposed to depend on the secondary metabolite structure and the number of hydroxyl groups in their molecule [58,161]. It was also noted that the pH value of the reaction medium influenced the capacity of phenolic compounds, in particular flavonoids, for interaction with metal ions [162,163,164]. Furthermore, the properties of these secondary metabolites within the flavonoid–metal complex were different from those of the original compounds [165]. It has been shown that the resulting flavonoid–metal complexes have a higher potential for ROS binding and preventing lipid oxidation, because their flavonoid components exhibit higher reactivity towards superoxide anion radicals [166]. Flavonoids are also known to have a reducing power towards the ions of Fe (III) and Cu (II), upon which the evaluation method of phenolic electron-donating activity is based [163,167].

Additionally, it has been found that free flavonoids present in plant cells (in the form of aglycones) have higher complexing potential towards Cd compared to their glycoside forms, which has a significant influence on plant metal tolerance [168,169]. It has been noted that the polyphenol complexation of Cd increases the transport of this metal in the plant organism [135]. It should be mentioned that flavonoid complexes with HM ions can have varied stoichiometry. Quercetin, for example, can form complexes with different metal-to-quercetin ratios and varying lipophilicity values [170]. Thus, flavonoid-dominated complexes are more lipophilic than free flavonoids, whereas complexes with high metal content are more water-soluble. The stoichiometry of forming complexes determines the nature of their interaction with the phospholipid bilayer and can influence its physicochemical properties, and protective and AO effects [156,171].

There is yet another mechanism responsible for the AO potential of polyphenols. It has been reported that as lipid peroxide (LOOH) reacts with metal ions, its structure can be damaged, which leads to the O–O bond cleavage and generation of lipid alkoxyl radicals, which initiate free radical chain oxidation. Meanwhile, phenolic compounds inhibit the process of LPO by trapping lipid alkoxyl radicals. The process is defined by the structure of the phenolic compound, the hydroxyl group numbers, and the positions in its molecule [172].

Some phenolic compounds can be the source of ROS themselves, exhibiting prooxidant properties, the same as enzymatic AOs [169]. Thus, the formation of superoxide anions was observed at neutral pH values as a result of the auto-oxidation of a number of phenolic compounds found in plants, such as gossypol [173]. It has also been reported that flavan-3-ols found in tea plants, which are characterized by their meta-5,7-dihydroxy-substituted A-ring and catechol or pyrogallol B-ring, can easily be oxidized. This mechanism was studied in great detail [174]. The auto-oxidation of catechins, catalyzed by the endogenous enzymes polyphenol oxidase and peroxidase, can take place even when the enzymes are inactivated or removed. It has been noted that the main structures promoting auto-oxidative processes epicatechin and epigallocatechin, two of the chief catechins found in green tea, possessed a combination of an extended conjugated system, fused rings, and at least one conjugated carbonyl group [175].

*Phenolic compounds and Cd*. The investigation of Cd effects on polyphenol accumulation and levels of the plant stress response is among the most dynamically growing areas of plant biology [132,176]. In most cases, stressed plants experienced a significant rise in the content of these secondary metabolites (Table 1).

However, it depends on plant species characteristics, vegetation regime (light, salinity, UV radiation, etc.), metal dose, and length of exposure. It has been reported that the polyphenol content in the plants of the *Asteraceae* family (wild chamomile—*Matricaria chamomilla*) increased under metal-induced stress [177].

Moreover, even low Cd concentrations boosted their capacity for flavonoid accumulation, as shown for several plant organisms [178,179], and increased the polyphenolic pool in the plants of *Vaccinium corybosum* (*Ericaceae* family) [176] and *Hypoxis hemerocallidea* [179]. A similar trend was observed for callus cultures of *Linum usitatissimum* [57]. As for the callus culture of *Camellia sinensis,* characterized by a high capacity for phenolic compounds production, its cultivation in the Cd-containing medium increased the pooling of phenolics, including flavans [180]. At the same time, an increase in the formation of lipid peroxidation primary products (conjugated dienes) was observed, accompanied by a drop in secondary product content (malondialdehyde), which suggests a rapid cellular response of the stressed tea plant [181].

It has been shown that various ‘defense’ agents are produced by plants as a response to Cd stress, and this process is species-specific. Grasses, for example, synthesize tricine, while legume plants produce compounds with sulfhydryl groups and cabbages—compounds with both phenolic and sulfhydryl groups [182].

**Table 1:** The effects of Cd on phenolic content (PC) in plants.

Plant Species	Plants’ Organs	Concentration Cd	PC	PC Level	Reference
*Matricaria chamomill*	Roots, shoots	4.5 and 16.5 mg Cd/kg soil	Total PC	Increase	[177]
*Malva parviflora*	Roots, shoots	40 μM Cd	Total PC, flavonoids	Increase	[179]
*Vaccinium corymbosum*	In vitro plantlets	50 and 100 μM Cd	Total PC, chlorogenic acid	Increase	[176]
*Linum usitatissimum*	Callus culture	15 мг/л Cd	Total PC	Increase	[57]
*Camellia sinensis*	Callus culture	25 мг/л Cd	Total PC, flavans	Increase	[180]
*Prosopis glandulosa*	Leaf	0.001 M Cd	Total PC	Decrease	[183]
			Gallic, vanillic, and caffeic acids, rutin, and kaempferol-3-O-glucosides	Increase	
*Withania somnifera*	Aboveground organs of seedlings	100 and 300 µM Cd	Total PC, flavonoids	Increase	[178]

In the case of Cd-induced stress, it is possible that the effect of secondary metabolite pooling in plants will be lacking. This can be explained by the plants’ use of other biochemical strategies, such as the synthesis of metallothioneins, which are known to be effective in bringing down Cd stress levels in various plant species [158,163]. Thus, the lack of polyphenol content increases in some *Prosopis glandulosa* Torr. plants (*Fabaceae* family) exposed to Cd stress can be the consequence of their AO system response damage due to high metal concentration effects, which is known to curb the plant’s capacity for the biosynthesis of these specialized metabolites [137,183].

In a number of cases, increased polyphenol accumulation in plant cells is accounted for by the activation of the key enzyme in their biosynthesis, phenylalanine ammonia lyase (*PAL*) (Figure 7). PAL helps to convert L-phenylalanine to cinnamic acid, one of the main precursors of all other phenolic compounds [58]. In HM-tolerant plants, for example, Cd stress led to an increase in PAL activity and the content of epidermal polyphenols, which act as chelators of metal ions and, through this, reduce the damaging effect of Cd. In some cases, the epidermal polyphenol content can decrease in the presence of Cd due to the disturbance of the AO system activity and the slowing down of the biosynthesis of new phenolic compounds [155]. The decrease observed in the pooling of quercetin, hydroxybenzoic and n-coumaric acids, together with PAL activity growth, suggests that secondary metabolites other than polyphenols are biosynthesized by the plants [183].

*Anthocyanins and stress.* Anthocyanins are among the most widespread flavonoids occurring in plants [184]. These compounds have AO properties, and since they are pooled predominantly in vacuoles close to ROS generation sites, they can rapidly enter into reactions with oxidative stress products. They, therefore, protect the plant against the inhibition of various physiological processes and promote plant adaptation to external factors [184,185].

It has been reported that anthocyanins are accumulated by various plant species exposed to HMs to an extent proportionate to pollutant emissions into the atmosphere [160,186]. It is believed that the biosynthesis of anthocyanins, which engage in oxygen radical detoxification processes, can be activated by the accumulation of such photolytic metabolites as superoxide anions, hydrogen peroxide, and singlet oxygen related to the activity of riboflavin, a photosensitizer, the content of which tends to increase under stress [160]. Anthocyanins are capable scavengers of superoxide radicals, which makes them effective endogenous AOs. They can reduce ROS toxicity and act as electron donors for peroxidase-mediated reactions, compensating for the lack of endogenous AOs, including ascorbic acid [187].

It has been demonstrated that HMs have an effect on the induction of anthocyanin biosynthesis. The most active metals in terms of inducing flavonoid accumulation are Cd and Cu. An increase in anthocyanin production was observed in wheat seedlings when exposed to Cd [147]. Additionally, the most stress-sensitive line exhibited a more intense induction capacity for anthocyanin biosynthesis, which is a prerequisite for successful stress coping. However, the AO properties of anthocyanins were eventually suppressed by high Cd concentrations, and their protective effect shrank.

**Figure 8:**
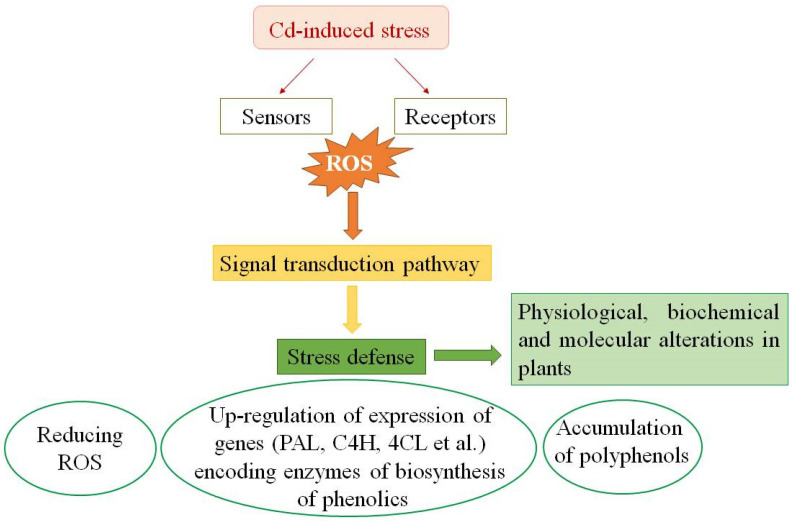
The role of phenolic compounds in protecting plants from the action of cadmium (Cd).

*Polyphenol biosynthesis genes and Cd.* The biosynthesis of polyphenols, including anthocyanin flavonoids, and the role of enzymes and genes responsible for their formation are well documented [188]. The latter include phenylalanine ammonia lyase (*PAL*), chalcone synthase (CHS), chalcone isomerase (CHI), anthocyanidin synthase (*ANS*), and others (Figure 8).

It has been shown that *MYB*, *bHLH*, *WRKY,* and a few other stress-responsive plant transcription factors regulate the expression of genes involved in the phenolic compound biosynthesis [189]. It has been reported that, under abiotic stress, anthocyanin accumulation in the top layer of plant epidermal cells is activated by the stress-induced ROS signal transduction with subsequent transcription of the regulatory factors that activate anthocyanin biosynthesis gene expression [178,190].

It is known that the molecular genetic basis of anthocyanin biosynthesis is common to various plant species, so the structural genes that code for the enzymes of this pathway are regulated by specific transcription factors. However, there are certain species-specific differences in their regulation mechanism [191,192], which allowed identifying anthocyanin biosynthesis patterns. Thus, an increase in the content of these secondary metabolites in stressed plants is accompanied by a higher transcript abundance of related genes encoding anthocyanin biosynthesis. This process depends on the stress type and intensity [193].

It has been shown that the upregulation of transcription of such genes as CHI, CHS, flavanone 3-hydroxylase (F3H), dihydroflavonol 4-reductase (DFR), and UDP-glucose: flavonoid 3-O-glucosyltransferase (UFGT) promotes anthocyanin biosynthesis in plants under metal-induced stress [147,189,194]. CHS gene activation in brassica napus (*Brassicaceae* family) under Cd stress was accompanied by the accumulation of anthocyanins, which act as metal chelators, reducing the damaging effect of the pollutant [195].

It has been established that the activation of genes inducing plant anthocyanin synthesis depends on the concentration of the HMs: it can only take place at low or moderate pollutant concentrations. At higher HM concentrations, the plants lose their ability to regulate genes effectively, which leads to their death [147]. It seems that the concentration dependence of this regulatory process is explained by strong oxidative stress caused by high metal concentrations and inhibiting plant capacity for the regulation of anthocyanin biosynthesis for protection and reparation purposes.

An increase in anthocyanin accumulation under Cd-induced toxic stress, together with the positive correlation between the pollutant content and flavonoid accumulation, suggests that their biosynthesis is a non-specific mechanism of plant adaptation to high metal concentrations, where the anthocyanin content can be used as an indicator of Cd contamination of the environment [196]. It has been assumed that anthocyanin synthesis activation is a stage of plant adaptation that determines the functioning of defense mechanisms under stress and promotes the acquisition of non-specific tolerance [187,195]. It has also been suggested that anthocyanins perform their protective function when stored inside vacuoles as vacuolar reserves [184,185]. Thus, according to the researchers working in this field of science, it can be concluded that anthocyanins are capable of additive interaction with other protective compounds founds in plants to eliminate the consequences of HM-induced stress and increase plants’ tolerance to their toxic effects.

## 7. Conclusions

Heavy metals are one of the most toxic compounds that are accumulated in significant quantities in the environment due to man-made human activity. Their impact leads to significant changes in the metabolism and viability of all inhabitants of our planet, from microorganisms to humans. The damaging effect of heavy metals is caused both by their direct action on the object, and is “mediated” by entering the body through food chains. In the latter case, it causes significant and even irreparable harm to the body, due to its “unpreparedness” and lack of protection systems.

The review provides information on the effects of HMs on metabolic processes and plant viability. The processes of inactivation and compartmentalization of these pollutants are reported. Much attention is paid to Cd as one of the most toxic representatives of HMs, the amount of which in the environment of many countries of the world has increased significantly in recent years due to anthropogenic human activity. It is this fact that explains the great attention paid by scientists of various specialties (biologists, ecologists, pharmacologists, and physicians) to the study of its effect on various biosystems.

Despite the significant progress in Cd research, many aspects of its action are still unclear. This concerns the mechanisms of receipt and inactivation of this pollutant in various representatives of the plant kingdom, which is important both for preserving their viability and productivity. Knowledge of physiological and biochemical processes, including under conditions of effective formation of reactive oxygen species in cells under the action of Cd, is necessary to develop a strategy for regulating plant adaptation, as follows from the material presented in the review. Phenolic antioxidants play an important role—they prevent the development of oxidative stress in plant cells caused by HMs, including Cd. By participating in the processes of complexation with pollutants, they prevent their absorption by plant tissues and reduce the negative consequences of anthropogenic activity. The stability of various plant objects, as well as their pharmacological value, depends on the regulation of the accumulation of polyphenols in plants, including their representatives, such as anthocyanins. Proceeding from this position, one of the important and relevant directions for further research may be the study of the accumulation and composition of these compounds of secondary metabolism, as well as the transcriptional and regulatory ability of plant tissues under the action of HMs.

All of the above allows us to conclude that due to the increase in the anthropogenic load on agrocenoses, further study of low-molecular-weight ligands, which include phenolic antioxidants, should be considered as an actively developing promising direction when conducting systematic monitoring of the elemental composition of plants. In addition, it plays an important role in improving the elemental status of plant products while maintaining its environmental safety.

## Data Availability

Not applicable.
